# Host-plant location by the Guatemalan potato moth *Tecia solanivora* is assisted by floral volatiles

**DOI:** 10.1007/s00049-017-0244-2

**Published:** 2017-08-29

**Authors:** Miriam Frida Karlsson, Magali Proffit, Göran Birgersson

**Affiliations:** 1 Department Plant Protection Biology, Swedish University of Agricultural Science, P.O. Box 102, 230 53 Alnarp, Sweden; 2 International Institute of Tropical Agriculture (IITA), 08 BP 0932 Tri Postal, Cotonou, Benin; 3 Centre d’Ecologie Fonctionnelle et Evolutive (CEFE), UMR 5175, CNRS, Université de Montpellier, Université Paul Valéry Montpellier, EPHE, 1919 route de Mende, 34293 Montpellier, France

**Keywords:** Attraction, Gelechiidae, Kairomone, Olfactometer, *Solanum tuberosum*

## Abstract

Insects locate their host plants using mainly visual and olfactory cues, generally of the exploited plant structure. However, when the resource is difficult to access, it could be beneficial to utilise indirect cues, which indicates the presence of reward (e.g., oviposition site or mate). In the present study, we investigated the host-plant location strategy of the monophagous Guatemalan potato moth *Tecia solanivora* (Lepidoptera: Gelechiidae). The larva of the moth feed exclusively on potato *Solanum* spp. (Solanaceae) tubers usually hidden below ground. Using electrophysiological and behavioural tests, we characterised the olfactory cues mediating the attraction of the moth towards their host plant. Odour blends were made to represent different potato structures: tubers, foliage, and flowers. Synthetic blends were created by combining potato-emitted compounds that were antennal active which showed positive dose–response. Attraction to these blends of compounds in relation to the mating status of males and females was tested in dual-choice Y-tube assays. Both males and females, virgin and mated, were attracted to a three-compound blend representing flower odour, while foliage and tuber blends attracted neither sexes. Oviposition bioassays indicated additionally that the floral blend enhances oviposition. We show that potato flower odour might indicate the presence of an oviposition site for the female and possibly an increased mating opportunity for both sexes. Our results provide one of the few examples of the use of floral odour as a reliable indicator of host and probably mating possibility for phytophagous insects exploiting a site spatially separated from the flower.

## Introduction

Host seeking behaviour in insects involves a sequence of actions, where the insect has to make a decision whether to continue with, or abandon the search. The insect acceptance of the host is dependent on the phenological and health status of the host plant (Masante-Roca et al. 2007; Dötterl et al. 2009), expressed partly as a release of specific volatile compounds (Jhumur et al. 2006; Tasin et al. 2007) or ratio of such compounds (Grison-Pigé et al. 2002; El-Sayed et al. 2008; Dötterl et al. 2009). Each plant structure has, moreover, a specific odour profile (Proffit et al. 2007; Karlsson et al. 2009). In many cases, the insect locates its host plant through the volatile cues emitted from the exploited plant structure (Bruce et al. 2005; Bruce and Pickett 2011). There are numerous examples, wherein floral volatiles attract species-specific pollinators via advertisement of the availability of reward for the insect (Dobson and Bergström 2000; Grison-Pigé et al. 2002; Raguso 2008). Odours released from foliage attract herbivores to feed or oviposit (Finch and Collier 2000; Proffit et al. 2011), providing a reliable cue for an appropriate site to the insect. Foliage volatiles may furthermore have an indirect effect of attracting natural enemies of herbivores (Pichersky et al. 2002; Tamiru et al. 2011), similar to flower odours that attract florivores, which deter pollinators (Theis 2006; Adler et al. 2012) or predators (Raguso 2004). Indeed, the exploited plant structure is not always easily accessible or visible and the use of an indirect cue providing information on resource quality could represent a highly beneficial strategy for insects. In a few cases, there is a spatial separation between the site of the plant structure releasing the attractive odour and the structure used by the herbivore insect. For example, the scent of leaves, which advertise flower presence for the weevil *Derelomus chamaeropsis* Fabricius (Coleoptera: Curculionidae) (Dufaÿ et al. 2003) or the plant- and soil-emitted semiochemicals, enhances oviposition by the clover root weevil *Sitona lepidus* Gyllenhal (Coleoptera: Curculionidae) on the clover leaves (Johnson et al. 2006). In the case of phytophagous insects, whose life cycle includes a soil-living larval stage, the use of an informative cue of host suitability for offspring development by parental insects living above ground during host-plant selection could be a highly beneficial strategy. Shoot-mediated suitability of below-ground plant parts may guide adult insects for oviposition (Johnson et al. 2006).

The Guatemalan potato moth*, Tecia solanivora* Povolny (Lepidoptera: Gelechiidae), is a good example of a phytophagous insect with spatially separated larval and adult feeding sites. The only known hosts of *T. solanivora* are the potato species, *Solanum tuberosum andígena*, *S. tuberosum tuberosum*, *S. chaucha,* and *S. phureja*, all of which are tuber-forming plants (Cifuentes and López-Ávila 2004; Cadena et al. 2005). It is not known where, or if, adults feed. The moth is distributed from Guatemala to Ecuador in Latin America and in the Canarias Islands, where it is a limiting pest on potato cultivation (Hilje 1994; Pollet et al. 2003). The number of Guatemalan potato moth trapped in potato field increases during the flowering stage of crop (Rodríguez et al. 1988; Barreto et al. 2003; Sánchez et al. 2005) and the main infestation occurs accordingly only after tuber formation and peak flowering (Torres et al. 1994; Barreto et al. 2003). In the field, females of *T. solanivora* deposit the majority of their eggs on the soil surface, close to the potato stem, so neither on the foliage nor on the flowers (López-Ávila and Barreto 2004; Karlsson et al. 2009). Likewise does the potato tuber moth *Phthorimaea operculella* (Zeller) (Lepidoptera: Gelechiidae) avoid ovipositing on the plants themselves but in the soil adjacent to host plants and not around non-hosts, which has taken as evidence that host-plant volatiles are involved in host-plant location (Traynier 1983).

So far, little is known about potato odour importance for *T. solanivora* location of mate and/or oviposition site. Interestingly, Bosa et al. (2011) reported that virgin females were more attracted to flower odour than to odours of the whole plant or to tubers, in an olfactometer test. As attraction to flowers among virgin female moths is generally associated with location of feeding sites and not of oviposition sites (Cunningham et al. 2006; Saveer et al. 2012), the role of potato flower odour in the host locating strategy of *T. solanivora* needs to be explored.

Potato emits volatile compounds that are species specific and others that indicate the quality and the phenological stage of the plants (Bolter et al. 1997; Agelopoulos et al. 2000; Karlsson et al. 2009, 2013). All potato plant structures, potato foliage, tubers, and flowers emit compounds, some of which are antennal active to *T. solanivora* (Karlsson et al. 2009). Semi-field trapping assays showed that the compound methyl phenylacetate (MPA) emitted from potato flowers in high amount and potato tubers in small quantities attracts *T. solanivora.* However, MPA in combination with a high concentration of 6-methyl-5-hepten-2-one (sulcatone), a compound found in tubers, reduces the attraction (Bosa et al. 2011).

The aim of the present study was to characterize the cues used by *T. solanivora* to locate its host. We hypothesize that, because tuber formation and flowering peak occur simultaneously in potato plants, potato flower odour could guide females to find an oviposition site. To test this hypothesis, behaviour assays were conducted to observe response of *T. solanivora* to synthetic blends, identified from and representing different potato plant structures. The mating status influence in the behaviour was tested as well as the importance of individual compounds in the most attractive blend.

## Materials and methods

### Experimental insects

A laboratory colony of the Guatemalan potato moth *T. solanivora*, originating from the Colombian Corporation for Agricultural Research (Corpoica), was established. The colony was interbred regularly with wild Colombian moths and was reared on potato tubers in containment facilities at SLU, Sweden. Insects were maintained in 60 ± 10% RH and 18 ± 2 °C under a photoperiod of L16:8D. Adults for electrophysical experiment were kept in Plexiglas cages (33 × 33 × 33 cm^3^) and fed with 10% honey solution, whereas adults for the olfactometer assays were fed only with water 24 h before experiments.

### Electroantennography

Synthetic compounds (Table 1) were tested in a dose–response function with electroantennographic recording technique (EAG) over four orders of magnitude: 0.01, 0.1, 1 and 10 μg μl^−1^. The compounds were diluted in hexane (99.9% Lichrosolve, Merck KGaA, Darmstadt, Germany). Identification and quantification of potato, *Solanum tuberosum*, var. Princess, odour were predominantly done in a previous study (Karlsson et al. 2009). Those analyses, besides combined gas chromatography and electroantennal detection (GC-EAD), revealed 17 compounds in potato headspace that are antennal active for *T. solanivora*. Additional collection from the same plant species and variety, tested with GC-EAD on females, showed that benzaldehyde, 1-octen-3-ol, 3-octanone, and 2-phenylethanol, are also antennal active. Thus, synthetic versions of these four compounds were here tested alongside the previously identified compounds. Female insects, aged 2–3-day post emergence and 12–24-h post mating were used for the assay. Unsexed pupae were left in a cage to allow adults to emerge and mate. For the first 30 min after the onset of the photophase, any copulating pairs were selected and gently transferred to a new case. Males and females were separated into different cages after a single complete mating event. Antennae were cut at the base and mounted with electroconductive gel (Cefar, Lund, Sweden) in a forked antenna holder (Syntech Equipment and Research, Kirchzarten, Germany) and continuously exposed to charcoal-filtered and humidified air (1.5 l min^−1^). The odour stimuli were prepared by applying 10 μl of each compound and at each dose on pieces of filter paper (0.5 × 2 cm), inserted in a Pasteur pipette. A puffing device (Syntech stimulus controller CS-55) delivered a 0.5-s long air puff with a flow of 1 ml sek^−1^ through the Pasteur pipette and into the humidified airstream passing over the antenna. Each antenna was exposed to a series of stimuli, within one concentration level, that were presented in random order, to avoid sphericity. The stimuli series always started and ended with blank, hexane, and female pheromone component (*E*)-3-dodecenyl acetate, [(*E*)-3-12:Ac) (Pherobank, Wageningen, The Netherlands >99.6%] (Nesbitt et al. 1985) as the reference, since this compound had shown reproducible EAG responses. Each stimulus was presented at least 1 min after the previous stimulus to avoid adaptation of the antenna. The maximum amplitude of the EAG responses was recorded and analysed with the EAG-adapted software (Syntech, EAGPro ver. 2.0).

**Table 1:** Synthetic chemical compounds used for electroantennal- and behavioural assays

Compound name	CAS-nr^a^	Brand	Purity %
Benzaldehyde	100-52-7	Fluka	99.5
δ-Elemene	20307-84-0	Gift^b^	>75
δ-Cadinene	483-76-1	Florida chemical	85.5
α-Caryophyllene	4586-22-5	Sigma Aldrich	>85
β-Caryophyllene	87-44-5	Fluka	98.5
α-Copaene	3856-25-5	Sigma Aldrich	>90
α-Cubebene	17699-14-8	Gift^b^	>75
Decanal	112-31-2	Sigma Aldrich	98
(*E,E*)-α-Farnesene	502-61-4	Bedoukian	>95
(*E*)-β-Farnesene	18794-84-8	Bedoukian	90
Germacrene D	23986-74-5	Gift^b^	>75
Methyl phenylacetate	101-41-7	Sigma Aldrich	99
β-Myrcene	123-35-3	ICN Biomedicals	90
Nonanal	124-19-6	Fluka	95
3-Octanone	106-68-3	Fluka	Purum
1-Octen-3-ol	3391-86-4	Acros AG	98
Phenylacetaldehyde	122-78-1	Sigma Aldrich	95
2-Phenylethanol	60-12-8	Merck-Schuchardt	98
Sabinene	3387-41-5	Fluka	98.5
Sulcatone	110-93-0	Sigma Aldrich	99
Tetradecanal	124-25-4	unknown	>70

^a^CAS, Chemical Abstracts Service number

^b^Gift from Prof. Anna-Karin Borg-Karlsson, KTH Stockholm, Sweden

### Y-tube olfactometer assay

A behavioural assay was set up to compare attraction to synthetic blends representing potato flower, foliage, and potato tuber, against control (hexane or potato tubers). Preference test between potato tubers and the most attractive blend was thereafter performed. Two-to-four-day-old males and females, both mated and virgin, were tested in olfactometer bioassays. Virgin adults were prepared by separating sexes at the pupal stage, using the location of the genital pore as a differentiation criterion (Rincón and López-Ávila 2004) and placing them thereafter in separate cages. Mated insects were tested 12–24-h post mating. Prior to each assay, moths were allowed to acclimatize for 12 h, individually separated in glass tubes (∅3 cm × 12.5 cm) in the bioassay room. Each moth was used only once and was not exposed to odour sources before the bioassay.

Synthetic blends for Y-tube olfactometer assays were made of synthetic compound prepared out of compounds that generated activity in the *T. solanivora* antenna and that also showed a significant positive correlation between dose and response in the EAG analysis. The selected compounds were mixed into three blends representing potato odours from flowers, foliage, and tubers, respectively (Table 2). The blends represented, in this way, potato odour, as we assume the moth would perceive the plant. The composition of the blends, amount and ratio, was based on compound emission from potato flowers, foliage, and tubers and adjusted to workable quantities (Karlsson et al. 2009). The synthetic compounds were diluted in hexane (99.9% Lichrosolve, Merck KGaA, Darmstadt, Germany), which alone also constituted the control. A cotton wick was inserted in a Teflon tube (∅2 mm and 30 mm long), which was thereafter inserted in a 2-ml vial. The synthetic treatments and the control were dispensed into vials and released through the cotton wicks. Release rates of the treatments were set by the evaporation rate of the carrier solvent, hexane. During the assay, vials were placed separately in 1.5-l glass chambers. One treatment consisted of tubers of potato, *Solanum tuberosum* var. Princess. One kilogram of tubers was placed inside the glass chamber. Each chamber was connected with Teflon tubes to one of the two 20-cm-long branches of the Y-tube shaped olfactometer that converged into a 20-cm-long common arm (∅35 mm). Moistened and charcoal-filtered air was pushed through each glass chamber, which delivered the control and the treatment at 0.2 m s^−1^ into the Y-tube olfactometer. Tests were conducted at scotophase condition with room temperature ranging between 17 ± 2 °C and RH 70 ± 5%. A 1 W red-light LED lamp allowed for behavioural observation.

**Table 2:** Foliage, tuber, and flower blend composition in *T. solanivora* behavioural assays, representing measured potato plant emission [ng plant^−1^ h^−1^]

Foliage blend	ng	Tuber blend	ng	Flower blend	ng
β-Caryophyllene	2000	Decanal	80	Methyl phenylacetate (MPA)	8000
β-Myrcene	400	3-Octanone	80	Phenylacetaldehyde (PAA)	40
(*E,E*)-α-Farnesene	400	1-Octen-3-ol	80	2-Phenylethanol (PE)	120
(*E*)-β-Farnesene	200	Nonanal	80	*MPA*	
Phenylacetaldehyde	40	Methyl phenylacetate	40		
2-Phenylethanol	40	Benzaldehyde	40	*PAA*	
Nonanal	40	Sulcatone	20		
		Phenylacetaldehyde	20	*PE*	
		2-Phenylethanol	20		

Moths were placed individually at the entrance of the common arm in the Y-tube. The moth was able to walk in the tubes and make minor flights. The position of the moth was recorded after 10 min. A response was scored when the insects walked further than two-thirds into one of the test arms and remained in that arm until the end of the test period. No choice was noted if the moth remained in the common arm. The position, left and right arm, of the tested treatments was changed randomly and noted. During the time, the insects spent in the Y-tube their behaviour were observed and recorded. The olfactometer were rinsed with soap and ethanol after every five moth tested and burned in a 300 °C hot oven overnight (8 h). We observed and registered behaviours such as probing with the ovipositor, proboscis extension, and insect motion in the tube.

### Oviposition assay

An assay was conducted to examine the effect of the most attractive blend on oviposition. One pair of 2–3-day-old male and female was first placed together in a 12.5-cm-long glass tubes (∅ 30 mm) in the beginning of the photophase. Pieces of fabric (50 × 50 mm) were fastened with rubber bands over the openings of each end of the tubes. These pieces of fabric closed the tube and served in the same time as an oviposition substrate for the moth that does not lay eggs on very smooth surfaces as the glass. Mating pairs were selected and placed in a humidified airstream, 0.2 m s^−1^, with an addition of either the solvent, hexane, or the flower blend that were used in the Y-tube bioassays described above. The release rate of the solvent and thereby of the compounds was controlled as described in the olfactometer assay. The number of eggs laid on the fabric was counted in the beginning of the following scotophase. The oviposition per female was counted once and 100 females were tested per treatment.

### Statistical analyses

Dose-dependent response in electroantennographical assays was calculated by first subtracting the amplitude of the EAG response of the solvent from the EAG response of each compound. This was followed by dividing the stimulus response by the mean of the responses to the reference (0.1 μg (*E*)-3-12:Ac) applied before and after the test stimulus, achieving a relative response. The residuals of the variables were normally distributed for most responses (*P* > 0.05 D’Agostino–Pearson normality test) and for those, repeated measures ANOVA were done, followed by Tukey’s multiple comparison test, to analyse the response to the different doses. For responses that were not normally distributed, Friedmans nonparametric test and Dunn’s post test were used (Prism 5.0a, Graphpad Software).

To determine moth attraction in the olfactometer assays, data from each compound blend were arranged in a 2 × 2 contingency table. Using Fishers exact test, we compared the proportion of moths that chose the right olfactometer arm or the left when the tested odour was on the left vs. on the right (excluding the ‘non-responders). The null hypothesis, here, was that if the tested odour had no effect on moth behaviour (e.g., attraction or repulsion); the distribution of the moth between the left and right arms of the olfactometer should be the same when the tested odour is offered on the left and on the right arm of the olfactometer. The additional behaviours observed performed by the insect in the olfactometer were analysed in a 2 × 2 contingency test comparing the number insects with additional behaviours, with responding insects in each group (sex and mating status).

The effect of the floral blend on the number of eggs laid per female was tested with a generalized linear mixed model (glmm) with day as a random factor, under Poisson distribution using the package lme4 (Bates et al. 2014) in R (R Core Team 2014).

## Results

### Electroantennography dose-dependent response

**Fig. 1:**
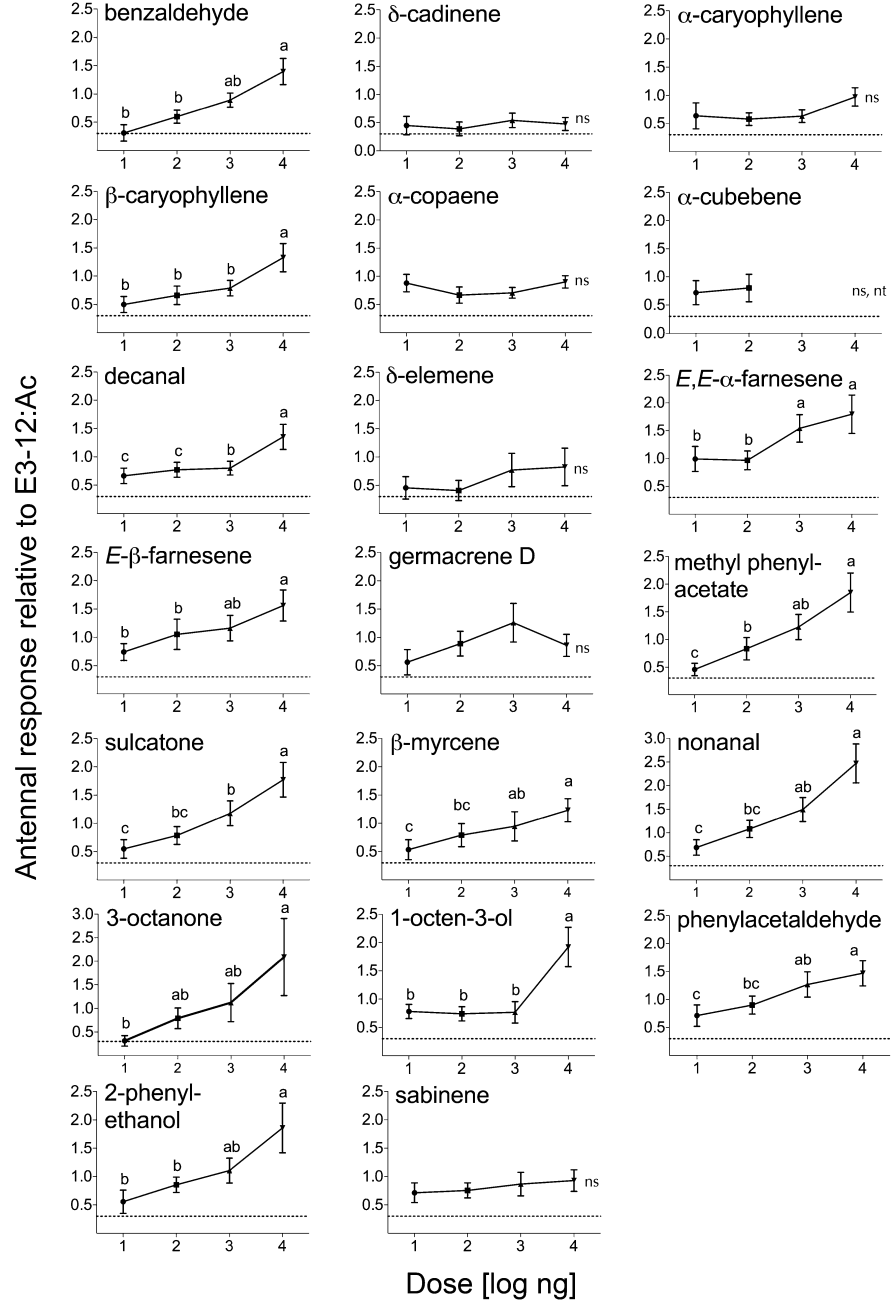
EAG dose response curves for *Tecia solanivora* females to synthetic plant volatiles. EAG amplitudes are control-adjusted and presented as relative response to the standard 0.1 μg (*E*)-3-12:Ac (*Tecia solanivora* pheromone). Doses tested were 0.01, 0.1, 1, and 10 μg μl^−1^. *ns* not significant, *nt* not tested

Out of the 21 compounds tested, 13 showed a significant positive dose-dependent response (Table 3). The compounds benzaldehyde, β-caryophyllene, decanal, (*E,E*)-α-farnesene, (*E*)-β-farnesene, methyl phenylacetate, β-myrcene, nonanal, 3-octanone, 1-octen-3-ol, phenylacetaldehyde, 2-phenylethanol, and sulcatone significantly increased the response with an increased dose, while several sesquiterpenes, e.g., α-caryophyllene, δ-elemene, and germacrene D, did not increased the EAG response with increased dose (Fig. 1; Table 3). Response to tetradecanal was low, hindering further analysis and usage of the compound. The 13 compounds, which elicited an enhanced response, caused by the quantity of the compounds on the filter paper, were selected to prepare synthetic blends for further bioassays.

**Table 3:** Dose effect of the synthetic chemical compounds on the EAG response of *Tecia solanivora* females

Compound name	*N*	*F* value^a^	*P* value
Benzaldehyde	10	9.192	0.0002
δ-Elemene	6	1.412	0.2781
δ-Cadinene	10	^b^	0.5296
α-Caryophyllene	10	0.211	0.0894
β-Caryophyllene	9	9.817	0.0002
α-Copaene	11	2.007	0.1341
α-Cubebene	9	^c^	0.4868
Decanal	16	11.57	<0.001
(*E,E*)-α-Farnesene	10	9.908	0.0001
(*E*)-β-Farnesene	10	7.541	0.0008
Germacrene D	10	1.984	0.1401
Methyl phenylacetate	15	^b^	<0.001
β-Myrcene	10	7.951	0.0006
Nonanal	11	^b^	<0.001
3-Octanone	8	^b^	0.0079
1-Octen-3-ol	11	15.07	<0.001
Phenylacetaldehyde	10	6.228	0.0024
2-Phenylethanol	10	5.576	0.0041
Sabinene	10	0.822	0.4934
Sulcatone	11	14.56	<0.001
Tetradecanal	2	n.a.	n.a.

^a^Repeated measures one-way ANOVA

^b^Non-parametric Friedman’s test

^c^Paired *t* test

### Olfactometer assays

**Fig. 2:**
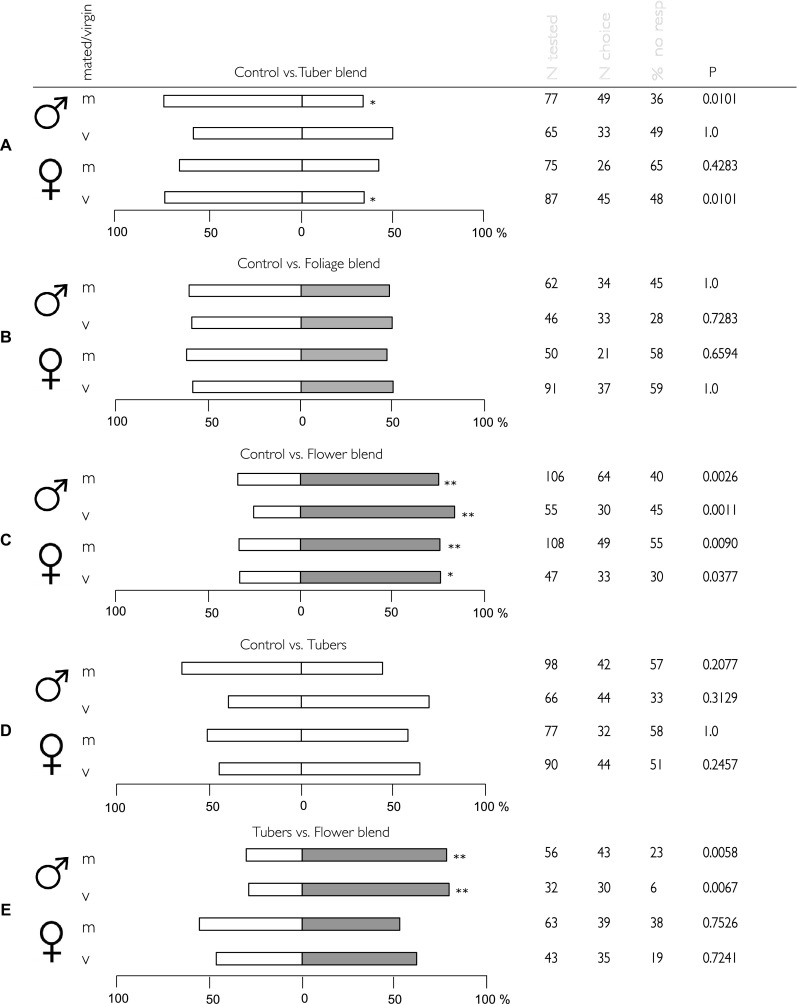
Response of male and female, virgin and mated *Tecia solanivora*, in Y-tube experiments when presented with odour blends (Table 2) vs control (**a**–**d**). **a** Tuber blend; **b** foliage blend; **c** flower blend; **d** tuber; and **e** tuber vs flower blend

In the first set of dual-choice bioassays, three synthetic potato blends (Table 2) were tested against a control of hexane alone. Whereas no significant difference in moth attraction behaviour was observed between tuber blend and control for virgin males and mated females, mated males and virgin female moths significantly avoided the tuber blend (Fig. 2a). In this dual choice, almost 70% of mated males and virgin females choose the control test tube. Neither male nor female moth significantly preferred foliage blend to control (Fig. 2b). *Tecia solanivora* males and females, both mated and virgin, were significantly attracted by the synthetic floral blend. Approximately 70% of the insects choose the arm of the olfactometer with the flower blend (Fig. 2c). In the dual-choice assay between potato tubers and the control, no significant directional trends for any of the tested groups were found (Fig. 2d). In the choice between flower blend and potato tubers, virgin and mated males were significantly more attracted to the floral blend than to the potato tubers, while females did not significantly chose between tubers or flower blend (Fig. 2e).

Analysis of observed moth behaviours in the olfactometer revealed that mated females displayed probing behaviour with the ovipositor more than the virgins in the experiments with potato tubers (*χ* ^2^ = 9.515, *df* = 1, *P* = 0.002), while in the other experiment, there was no difference in how often the ovipositor was extended (Table 4). Proboscis extension was only observed in about 1% of the tested insects.

**Table 4:** *Tecia solanivora* ovipositor extension during olfactometer assays in different treatment combinations

Treatment combination	Virgin (%)	Mated (%)	*P* ^a^
Tuber blend vs control	7	12	0.413
Foliage blend vs control	7	16	0.176
Flower blend vs control	10	20	0.19
Tubers vs flower blend	0	11	0.006
Tubers vs control	2	31	<0.001

^a^ *χ* ^2^ test

### Oviposition

The number of eggs laid per female differed between treatment with the synthetic three-component flower blend and the control (glmm, *χ* ^2^ = 287.21, *df* = 1; *P* < 0.001) and was higher in the presence of the flower blend [35.7 ± 2.9 (mean ± SE), *N* = 110] than in the control (23.7 ± 2.6, *N* = 105).

## Discussion

By combining electrophysiological recordings and behavioural assays, we characterised olfactory cues used by the Guatemalan potato moth to locate its host, potato. An attractive odour cue is emitted by potato flowers and corresponds to a blend of three structurally similar compounds, methyl phenylacetate, phenylacetaldehyde, and 2-phenylethanol. Oviposition was significantly higher when moths were held in an airstream of the three-component flower blend, than in the control. This result indicates that mated females distinguish oviposition sites by a compound blend emitted specifically by potato flowers and that flower odour is a cue for a preferred oviposition site. The divergence between the plant structure that is exploited by the insect (potato tubers) and the plant part with an attractive odour emission (potato flowers) is one of the very few examples, where spatially separated plant structures emit cues representing the presence of a suitable host in the vicinity (Dufaÿ et al. 2003; Johnson et al. 2006; Kehl et al. 2010).

Potato growth can be divided into phenological phases such as sprouts emergence and root growth followed by leaf development and tuberization. The development of tubers coincides with the flowering of the plant (Rodríguez-Falcón et al. 2006). Since *T. solanivora* larvae feed exclusively on tubers, this implies that the most suitable stage for the larvae is correlated with the flowering of the plant. Our results corroborate the “preference–performance” hypothesis, or “mother knows the best”, first proposed by Jaenike (1978), stipulating that females are usually attracted to plant species, plant structure, and/or phenological stage that offer a positive influence on the progeny performance (Masante-Roca et al. 2007; Proffit et al. 2007; Gripenberg et al. 2010). The adult lays eggs in the soil near the base of the plant and it is the first instar larvae that locate the potato tuber in the soil by geotaxis and no chemical cues influence its orientation (Camargo Gil et al. 2010). For larvae of *T. solanivora*, like most Lepidoptera larvae, survival depends of fast location of a food source and onset of feeding. Yet, neonates are exposed on the soil and their vulnerability is dependent on the oviposition choice of the adult.

Most electroantennal active compounds consistently showed a dose-dependent antennal detection by individual moths. Blends created based on these compounds might be considered the odour of potato as *T. solanivora* perceive the plant, since the blends do not contain the full bouquet of volatile compounds emitted by potato. The synthetic blends representing foliage and tuber odour were not attractive in our study. In fact, synthetic tuber blend was even significantly less preferred than the control. Our results for the foliage blend concur partly with earlier work that showed that *T. solanivora* selection of odour source in a 4-arm olfactometer did not differ between whole potato plant and the control, while odour of flower was selected the most (Bosa et al. 2011). Composition of the tuber blend, causing a discrepancy between behavioural responses to tuber odour, might imply that the blend was not completely representative of tuber odour, for the moth. An incorrect proportion of the compounds might have caused the avoidance observed here of the synthetic blend. An incomplete headspace collection, identification, and/or calculation of the release quantity might also account for the divergence in behavioural response between tubers and tuber blend.

Host-plant odours generally elicit attraction behaviour in Lepidoptera species while locating feeding sites (Browne 1993; Dobson and Bergström 2000), oviposition sites (Grant et al. 2007; Anfora et al. 2009), or mating encounter sites (Tasin et al. 2005). Currently, adult feeding behaviour of *T. solanivora* in the field is unknown, but it is unlikely that potato elicits attraction to feed. The Guatemalan potato moth has not been reported visiting flowers. In addition, potato flowers are nectarless and pollen is the sole reward for visiting insects. Most flowers in the genus *Solanum* are showy and sweet-scented (Buchmann and Cane 1989) and exclusively buzz-pollinated by bumble bees (Anderson et al. 1988) or by specialized solitary bee species from the Apidae, Colletidae, and Halictidae families that vibrate flowers to extract the pollen from the merged anther cones (Kessler et al. 2011). Hence, the attraction of *T. solanivora* to potato flower blend is probably an indicator of a suitable phenological stage, i.e., the formation of tubers, rather than a food source for the moth.

The three-component flower blend, comprising of methyl phenylacetate, phenylacetaldehyde, and 2-phenylethanol, was attractive to both females and males, virgin and mated. Our results suggest that these three biosynthetically closely related substances are important for the adult moth host finding (see Table 2 for structural formulae). Potato flowers close during the night, potentially reducing odour emission. When the sun rises, the flowers open again, concurrently with the onset of female calling. At sunrise, or during the onset of an artificial photophase, females start to call. This last for approximately 30 min. *Tecia solanivora* males and females are penumbras and are most active and fly around between 5 and 8 a.m. and to a lesser degree between 6 and 7 p.m. (Barreto et al. 2003). It is known that a combination of pheromone components and plant compounds, such as floral scent, can enhance male attraction in phytophagous insect species (Reddy and Guerrero 2004). The presence of host plants can also increase pheromone production and emission, and elicit earlier onset and prolonged calling behaviour (Ochieng et al. 2002; Srinivasan et al. 2003; Sadek and Anderson 2007). For virgin adults, flower compound might influence the mate-searching behaviour. Our results are comparable to those of previous studies on the influence of host plants on mate-searching of *Heliconius charithonia* L. (Lepidoptera: Nyphalidae) (Estrada and Gilbert 2010) and with female-calling behaviour in *Helicoverpa armigera* Hubner (Lepidoptera: Noctuidae) (Xiao et al. 2002). It is known that if mating occurs next to the host plant and if both sexes search the host place prior to mating, the probability to encounter a conspecific is increased, especially for monophagous insects (Browne 1993; Wickman and Rutowski 1999). We hypothesize that the same flower odour acts as an odour cue for virgin insects, attracting them to the potato plant in the morning, which indirectly enhances the probability of encounter.

Mated females of many phytophagous moth species have an increased behavioural responsiveness, than virgin females, to host-plant odour (Tingle et al. 1989; Odendaal and Rausher 1990; Masante-Roca et al. 2007; Saveer et al. 2012). For example, mated females of *P. operculella* and *Tuta absoluta* Meyrick (Lepidoptera: Gelechiidae) are more attracted to their respective host plants, potato and tomato, than virgin females (Arab et al. 2007; Proffit et al. 2011). We observed, on the contrary, that the general behavioural responsiveness for *T. solanivora* tended to be higher by virgin females than by mated females. Complementary bioassays are required to test this difference as well as differences between treatments. Our observed higher responsiveness by virgin females might as well indicate an adaptation to minimize the cost of remaining unmated for a long time (Bergman et al. 2011). Because mating occurs next to the host plant, the already-mated females have less need to search for suitable host and mating (Milonas et al. 2011). Searching might therefore decrease just after mating, and the insects stay close to the host to oviposit in the soil nearby. *Tecia solanivora* oviposition begins during the first scotophase after mating and continues for several subsequent days (Karlsson, pers. observ.; (Torres et al. 1994). In our olfactometer assay, mated females extended their ovipositor significantly more often than the virgin females, which indicates that the mated females were ready to oviposit during the time of the bioassay. Ovipositor extension and dragging of ovipositor over the surface to evaluate the suitability of the site is related to the assessment process of the host-plant surface as mechano- and chemosensory receptors are found in the ovipositor in Lepidoptera (Renwick and Chew 1994). For *T. solanivora* as well as *P. operculella,* the surface structure is important for oviposition and they will extremely rarely oviposit on a smooth surface like glass, but prefer a rough dry surface, such as soil or substrates that provide depressions (Fenemore 1988).

Our results show that both sexes of adult *T. solanivora* are attracted to their host plant by similar bouquet of compounds emitted by flowers. We conclude that mating close to the potato plant reinforces the chance of oviposition in/on/around the same plant, explaining the mated female attraction to flower odour, in search for an oviposition site. A possible explanation of the attraction by *T. solanivora* males is that potato flower odour can act in combination with the female sex pheromone, emitted at sunrise.

This study gives an interesting example of the complexity of host-plant selection strategies in phytophagous insects. Emission of some volatile compounds from potato flowers is ensuring the attraction of adults of both sexes to the suitable stage of the host plant for larval development. Females’ absence of behavioural discrimination between the odour of the structure emitting the attractive cues, i.e., flower, and the odour of the actual exploited structure, i.e., the tuber, indicates that several odour cues might be involved in host-plant selection. Similar to Theis (2006) and Kehl et al. (2010), we show that flower scents are not only important cues for chemical communication between plants and their pollinators, but can also attract phytophagous insects even if they or their offspring do not feed from these organs. Oviposition behaviour of *T. solanivora* can be compared with the tomato fruit fly *Neoceratitis cyanescens* (Diptera: Tephritidae), which does not oviposit on flowers or fruit but are attracted to these organs (Brévault and Quilici 2010). The significant attractiveness of the flower blend in the olfactometer, as well as weak luring capacity by the flower blend in the field (Karlsson 2011), suggests that *T. solanivora* uses odour for long-range attraction, but that it may not be sufficient to elicit landing or acceptance on flowers, tubers not foliage in the short range. Future experiments with combination of volatile and visual cues from soil, potato tubers, and flowers, together with combination of flower compounds and pheromones may enhance our understanding of the intriguing host-plant selection behaviour of the Guatemalan potato moth.
